# Transient hypothyroxinemia of prematurity and its risk factors in an extramural neonatal intensive care unit

**DOI:** 10.20945/2359-3997000000360

**Published:** 2021-04-27

**Authors:** Ruchi Rai, Dharmendra Kumar Singh, Bhanu Kiran Bhakhri

**Affiliations:** 1 Super Speciality Pediatric Hospital and Postgraduate Teaching Institute Department of Neonatology Noida UP India Department of Neonatology (Maternal Reproductive Health), Super Speciality Pediatric Hospital and Postgraduate Teaching Institute, Noida, UP, India.; 2 Super Speciality Pediatric Hospital and Postgraduate Teaching Institute Department of Pediatrics Noida UP India Department of Pediatrics, Super Speciality Pediatric Hospital and Postgraduate Teaching Institute, Noida, UP, India.

**Keywords:** Congenital hypothyroidism, delayed TSH elevation, preterm infant, transient hypothyroxinemia of prematurity

## Abstract

**Objective:**

Thyroid functions in preterm newborns may be altered in the first week of life. Hypothyroxinemia has been commonly reported in these babies, which could be due to the immaturity of the hypothalamic pituitary thyroid axis or acute illness. It could have a long-term impact on the developing brain of these babies. We conducted this study to estimate the incidence of transient hypothyroxinemia of prematurity (THOP) and to determine its risk factors.

**Materials and methods:**

We analyzed thyroid stimulating hormone (TSH) and free T4 levels of 64 preterm neonates admitted in the neonatal intensive care unit. TSH and free T4 levels were measured in the first week and then at 14-21 days of life to estimate the incidence of THOP and determine its risk factors. We also estimated the incidence of congenital hypothyroidism (CH) and delayed TSH elevation in CH. Risk analysis was conducted using simple and multiple logistic regression, and numerical data was compared using the Mann Whitney U test and t test.

**Results:**

THOP was seen in 25% of the preterm babies. Caesarean delivery, presence of one or more morbidities, mechanical ventilation, birth weight ≥ 1,500 g, and gestational age ≥ 32 weeks were identified as risk factors for THOP based on simple logistic regression. In multiple regression, mechanical ventilation and gestational age ≥ 32 weeks were significantly associated with THOP. CH was seen in 2 (3.1%) babies, and 1 of these cases had delayed TSH elevation.

**Conclusion:**

Thyroid abnormalities are common in preterm admitted neonates. Mechanical ventilation is an independent risk factor for development of THOP.

## INTRODUCTION

Thyroid function in sick preterm babies is poorly understood. Many of these babies have low thyroxine levels in the first week of life. They can also have congenital hypothyroidism (CH) without any thyroid stimulating hormone (TSH) elevation in the first week, and CH can therefore be missed in the first screen. TSH elevation is delayed in such babies and can be detected only if a repeat screening is done later at 2-4 weeks (1,2). Alteration in the thyroid homeostasis in these babies may have far-reaching consequences on their developing brain (3).

Transient hypothyroxinemia of prematurity (THOP) is the most common thyroid dysfunction in preterm infants. This may be due to withdrawal of maternal T4 transfer, impaired production of T4-binding globulin, immaturity of the hypothalamic pituitary thyroid (HPT) axis, impaired synthesis, decreased iodine stores, use of drugs such as dopamine and steroids, inadequate nutrition, and non-thyroidal illnesses (4,5). THOP is characterized by transient, low levels of circulating thyroid hormone, T4/free T4 (FT4) with normal TSH levels. It usually resolves by 2-3 weeks of life with maturation of the HPT axis.

Evidence from past studies indicates that there is a higher incidence of CH in preterm infants, often detected only upon rescreening at 2-4 weeks of life (1,6). The diagnosis of CH in sick preterm newborns is extremely challenging, and interpreting the thyroid function can also be difficult in these babies. Despite several guidelines being published regarding screening for CH, there is still a lack of clarity regarding cutoff values for diagnosis of CH, optimum screening strategies, management, and outcome in preterm babies (7–9).

Many studies have been conducted on THOP and rescreening for diagnosing CH in preterm babies worldwide, but there is a lack of any such data from India. Therefore, we conducted this study to estimate the incidence of THOP and to determine its risk factors. The study also aimed at estimating the incidence of CH with delayed TSH elevation in preterm babies.

## MATERIALS AND METHODS

This was a prospective cohort study conducted in a neonatal intensive care unit (NICU) of a tertiary level teaching institute. Ours is a 20-bed level III unit that caters to extramural babies. The study was conducted over a period of one year from April 2019 to March 2020. The study was approved by the institutional ethics committee (IEC). Informed consent was obtained from the mother and/or father of the baby.

The **primary objective** of the study was to estimate the incidence of THOP and to determine the risk factors in the preterm babies admitted to the NICU.

The **secondary objectives** were as follows:

- To estimate the incidence of CH with delayed TSH elevation in admitted preterm babies.
- To determine the correlation between TSH and FT4 with the birth weight and gestational age of preterm newborns.

**Inclusion criteria:** Neonates admitted to the NICU were eligible for inclusion in the study if they fulfilled all the following criteria:

- Gestational age < 37 weeks
- Admitted at ≤ 7 days of life
- Hospital stay ≥ 7 days

**Exclusion criteria:** Babies who did not complete the study because of death, leaving against medical advice, or loss to follow-up after discharge were excluded.

All newborns admitted in the NICU over the period of one year who fulfilled the inclusion criteria were enrolled in the study. Their details were provided in a pro forma that included the demographic profile such as birth weight, gestational age, and gender and medical details such as duration of hospital stay, morbidities, and administration of dopamine. The babies were stratified into appropriate for gestational age (AGA), small for gestational age (SGA), and large for gestational age (LGA) (10). The newborns were managed according to the standard treatment guidelines. It is a unit policy to send 2 mL of venous sample (**Sample I**) for TSH and FT4 at 48-72 hours of life for all babies admitted within 24 hours of life and on the day of admission for babies admitted after 48 hours of life. A repeat venous sample (**Sample II**) is sent for TSH and FT4 at 14-21 days of age for all preterm babies. The first samples are TSH (1) and FT4 (1), and the repeat samples are TSH (2) and FT4 (2). The samples are analyzed using the enzyme-linked automated fluorescent immune assay (ELFA) technique (VIDAS, BIOMERIEUX).

Reference values for serum FT4 and TSH in the first week of life are as follows (11):

**Table:** 

Gestational age (weeks)	FT4 (pmol/L)	TSH (mIU/L)
25-30	6.4-28.31	0.2-20
31-36	16.7-56.63	0.7-20

TSH was considered elevated if the levels were > 20 mIU/L in the first week and > 10 mIU/L after the first week. The FT4 levels were considered low if the values were < 16.7 pmol/L in babies with a gestational age of 31-36 weeks and < 6.4 pmol/L for gestation of 25-30 weeks (11). THOP was defined as transient low levels of FT4 without elevation of TSH levels (5).

CH was diagnosed as per cutoffs for TSH, and CH with delayed TSH elevation was considered when TSH (1) was normal but TSH (2) was elevated.

Statistical analysis: Statistical analysis was performed using Epi info 7 and SPSS version 27. The normally distributed numerical data were analyzed by t test. The Mann Whitney test was used to compare the numerical data, which was not normally distributed. The Wilcoxon rank sum test was used for the paired data, and Pearson correlation was used to assess the correlation. Risk analysis was conducted using simple and multiple logistic regression.

## RESULTS

A total of 76 babies fulfilled the inclusion criteria. Out of these, 2 babies died, 2 left against medical advice, and 8 were lost to follow-up and could not complete the study. A final analysis of 64 babies was conducted. The comparisons of the demographic and clinical characteristics of the babies with and without THOP are given in Table 1. Out of the total babies, 34 (53.1%) were admitted on day 1 of life.

**Table 1:** Demographic and clinical characteristics of the study population

Characteristics	THOP N = 16	No THOP N = 48	p value
Male (%)	51	56	0.6
Vaginal delivery (%)	62.5	64.6	0.8
Birth weight (g) Mean (SD)	1,637 (437)	1,363 (369)	0.01*
Gestation (weeks) Mean (SD)	33.1 (3.3)	31.1 (2.9)	0.02*
Hospital stay (d) Mean (SD)	21.5 (10.5)	23.1 (13.4)	0.6
RDS N (%)	3 (18.7)	9 (18.7)	1
Sepsis N (%)	5 (31.3)	14 (29)	0.8
Mechanical ventilation N (%)	9 (56)	14 (29)	0.05
Dopamine N (%)	1 (6.7)	4 (8.3)	0.8
FT4 (1) (pmol/L) Mean (SD)	11.1 (4.4)	20.5 (7.1)	<0.01*
FT4 (2) (pmol/L) Mean (SD)	16.4 (4.7)	18.9 (4.6)	0.06
TSH (1) mIU/L Median (IQR)	2.2 (1.2,3.1)	3.9 (2.4,5.7)	0.01*
TSH (2) mIU/L Median (IQR)	3.4 (2.3,15)	4.1 (2.2,6.1)	0.5

* Significant.

THOP: transient hypothyroxinemia of prematurity; SD standard deviation; IQR: inter quartile range; RDS: respiratory distress syndrome; FT4: free T4; TSH: thyroid stimulating hormone.

THOP was observed in 16 (25%), [confidence interval (CI); 15%, 37.4%] babies, and the median TSH values in babies with THOP was significantly lower than in the babies without THOP (p = 0.016) (Table 1). Out of a total of 64 babies, 42 had one or more morbidities, and 22 did not have any morbidities. All 22 babies without morbidities were very low birth weight (VLBW) babies and were admitted only because of their VLBW. A comparison of the characteristics of babies with and without morbidities is given in Table 2. Presence of one or more morbidities, mechanical ventilation, birth weight ≥ 1,500 g, and gestational age ≥ 32 weeks were identified as significant risk factors. The significant risk factors were further analyzed by multiple regression to eliminate the confounding effect of one factor on another. Mechanical ventilation and gestational age ≥ 32 weeks were independently associated with higher risk of THOP (Table 3). Birth weight was not analyzed by multiple regression as it is strongly associated with gestational age. The FT4 levels returned to normal in 11 (68.7%) out of the 16 babies with THOP. We did not measure FT4 levels beyond 2-3 weeks.

**Table 2:** Comparison of demographic and clinical characteristics of babies with and without morbidity

Characteristics	Babies with morbidities N = 42	Babies with no morbidity N = 22	p value
M:F	1.4:1	1:1.7	0.07
Vaginal delivery (%)	59.5	72.7	0.29
Birth weight (g) Mean (SD)	1,508 (440)	1,286 (270)	0.03*
Gestation (weeks) Mean (SD)	31.5 (3.3)	31.7 (2.9)	0.8
Age at admission (d) Mean (SD)	2.5 (1.9)	2 (1.5)	0.2
Hospital stay (d) Mean (SD)	24 (14.1)	20.2 (9.2)	0.2
FT4 (1) (pmol/L) Mean (SD)	16.4 (7.7)	21.5 (6.7)	0.01*
TSH (1) mIU/L Median (IQR)	3.1 (1.5,5.3)	3.4 (2.4,4.8)	0.47

* Significant.

SD: standard deviation; IQR: inter quartile range; FT4: free T4; TSH: thyroid stimulating hormone.

**Table 3:** Logistic regression analysis of risk factors for THOP

Characteristics	Unadjusted OR (CI)	adjusted OR(CI)	p value^a^
Male sex	0.77 (0.24, 2.42)	–	
LSCS	1.09 (0.33, 3.53)	–	
BW ≥ 1,500 g	3.8 (1.14,12.64)	–	
GA ≥ 32 week	5.57 (1.4, 22.1)	8.6 (1.8, 40.6)	0.006
MV	3.12 (0.9, 10.03)	6.33 (1.1, 34.1)	0.03
Morbidity	1.8 (0.5, 6.4)	0.83 (0.15, 4.6)	0.8
Dopamine	0.73 (0.07, 7.08)	–	

a Adjusted OR.

THOP: transient hypothyroxinemia of prematurity; OR: odds ratio; CI: confidence interval LSCS: lower segment caesarean section; BW: birth weight; GA: gestational age; MV: mechanical ventilation. Unadjusted OR: univariate logistic regression.

Adjusted OR: multiple logistic regression.

Table 4 provides a comparison of the TSH and FT4 levels among the different groups. The medians of both values of TSH were lower and FT4 levels were higher in SGA preterm babies, but there was no statistically significant difference between the groups.

**Table 4:** Comparison of TSH and FT4 levels across stratified groups

	AGA (N = 44)	SGA (N = 17)	LGA (N = 3)	p value^a^
TSH (1) (mIU/L)	3.2 (1.5,4.7)	2.8 (2.1,5.6)	4.5 (2.9,9)	0.57
TSH (2) (mIU/L)	4.6 (2.1,6.2)	3.4 (2.5,4.1)	2.6 (2.4,5)	0.44
FT4 (1) (pmol/L)	18.6 (11.1,22.5)	22.7 (14.1,25.6)	13.9 (7.6,15.9)	0.15
FT4 (2) (pmol/L)	18.5 (15.6,22)	19.4 (16.1,20.8)	12.9 (10.3,17.2)	0.18

All values expressed as median (inter quartile range)

a Mann-Whitney U test.

AGA: appropriate for gestational age; SGA: small for gestational age; LGA: large for gestational age.

CH was diagnosed in 2 (3.1%) (CI; 0.4%, 10.8%) babies, 1 baby had elevated TSH on the first sample, and 1 baby was diagnosed on repeat sampling. The baby with CH had normal FT4 levels in the first week, as well as at the second screening. The baby with CH with delayed TSH elevation had low FT4 levels at the first screening, which normalized at the second screening.

We did not find any correlation between the birth weight and TSH (1) and FT4 (1) values of the study population, the correlation coefficient r being 0.04 and 0.01, respectively. Similarly, no correlation was found between the gestational age and TSH (1) and FT4 (1) values, the correlation coefficient r being 0.02 and 0.1, respectively. The Wilcoxon rank sum test for the paired values of TSH and FT4 values showed no significant difference, z = −1.8, p = 0.06 for TSH (1) and z = −0.03, p = 0.97 for FT4 (1).

The 2 infants diagnosed with CH were started on treatment. No treatment was started for transient hypothyroxinemia.

## DISCUSSION

We studied the TSH and FT4 levels in the first and third weeks of life in preterm babies admitted to the NICU. THOP was found in 16 babies, and CH was seen in 2 babies.

The strengths of the study are i) it was a prospective study and ii) we screened the babies for both TSH and FT4 levels simultaneously, which is the ideal screening approach. Limitations include i) the babies were all extramural babies, the majority having no antenatal records, meaning we did not have maternal thyroid status details, and ii) we did not measure free T3 levels, which could have added more information.

THOP was seen in 16 babies, an overall incidence of 25% among the preterm babies. Studies have reported THOP to be present in 15%-30% of preterm babies (9,12). Chung and cols. reported 23% of preterm babies having THOP (1). A higher incidence (85%) of THOP has been reported in very preterm babies (13). Mechanical ventilation and gestational age ≥ 32 weeks were associated with presence of THOP in our study. Herring and cols. identified mechanical ventilation, gestational age, and dopamine administration as risk factors for THOP (14). Another study found the prevalence of THOP to be associated with male gender and dopamine administration, but gestational age and birth weight were not risk factors (15). Huang and cols. found THOP to be related to brain ultrasound anomaly, neonatal illness, and lower Apgar score at 1 minute (16). Association of higher gestational age with THOP in our study could be because all babies with gestational age ≥ 32 weeks, staying for ≥ 7 days, were those with morbidities, but the neonates with gestational age < 32 weeks remained admitted even if they were well and had no morbidities. Acute illness in babies, such as respiratory distress syndrome (RDS), has been shown to be associated with lower levels of T4, FT4, and T3 in the first week of life (2,6,17). The FT3 and FT4 levels have been found to be lower during the period of critical illness. As the low FT4 levels were seen to be associated with morbidity in our study as well, this could be due to the euthyroid sick syndrome. The factors responsible for low thyroid hormone in the first week resolve by the third week, as there is onset of maturity of body functions and recovery from acute illness, leading to a rise in the FT4 levels (18,19). TH is considered a benign and transient condition. However, as the early weeks of life are crucial for the developing brain, especially in preterm babies, there have been concerns regarding the effect of low thyroxine levels on the developing brain (20,21).

There are contradictory results from different studies that have been conducted on long-term follow-up of preterm babies with THOP. Many studies have found adverse effects in the later life of these newborns, such as cerebral palsy and impaired cognitive functions (3,22). On the contrary, other studies did not find any association between THOP and neurodevelopmental outcome (23,24). Scratch and cols. showed that paradoxically, higher postnatal FT4 levels in very preterm infants resulted in poorer neuropsychological development in childhood (25). Thyroxine supplementation of infants born at < 30 weeks gestation has been shown to promote better neurological outcome later in life (26). Supplementation with levothyroxine has been shown to cause adverse events such as circulatory collapse in some studies (27).

A rare scenario of central hypothyroidism might also have similar presentation of low FT4 and normal/low TSH. A thyrotropin-releasing hormone stimulation test might be the only way of differentiating between the two entities (5).

In our study, 2 babies were diagnosed with CH, and one of them had delayed TSH elevation (1.5%). Variable incidences of CH ranging from 1% to as high as 18% have been reported in preterm babies (28). The studies varied in terms of not only gestation and birth weight of neonates included, but also the TSH and FT4 cutoff levels. Hemmati and cols. studied 67 critically ill preterm babies, of which 5 (7.4%) showed delayed TSH elevation (19). Chung and cols. studied 105 preterm babies < 32 weeks of gestational age; 13 babies had CH, of which 8 (7.6%) neonates had CH with delayed TSH elevation (1). The European Society for Pediatric Endocrinology recommends rescreening for CH in all preterm babies (9). The Indian Society for Pediatric and Adolescent Endocrinology recommends that high-risk neonates, such as preterm, low birth weight (LBW), VLBW, and ill neonates admitted to the NICU, as well as multiple births (especially same-sex twins), should have a second screening at 4 weeks (or at 2 weeks of age if discharged early) (7).

Delayed TSH elevation, not due to CH, may be seen in some high-risk babies, which occurs at 2-6 weeks and resolves at 6-10 weeks. This has been named atypical hypothyroidism or transient elevation of TSH. This can be due to recovery of illness, induced suppression of the HPT axis, iodine deficiency or excess of iodine levels, maternal antithyroid drug, or maternal autoantibodies (5).

We did not find any significant difference in the median TSH and FT4 values of different groups of the preterm neonates and between the paired samples in each group. Our findings are consistent with the findings of other studies, where the authors did not find any difference in the FT4 and TSH levels of preterm SGA babies (29,30). Liu and cols. found significantly higher median TSH and FT4 levels in SGA preterm babies, but the levels were within normal limits (31). Higher TSH and FT4 levels have been reported in other studies as well (32). In a population-based study from India, the TSH levels in the first week were found to be higher in male neonates, LBW babies, and those born by vaginal delivery (33). Thyroid dysfunction reported more commonly in babies with LBW could be because of poor intrauterine nutrition, hypoxia, and acidosis.

We did not find any correlation between the gestational age and birth weight and the TSH and FT4 levels. Korkmaz and cols. found no correlation between the first TSH level and gestational age and birth weight, but an inverse relationship was found in the second and third samples (2). A significant correlation was found between FT3 and FT4 with the gestational age and birth weight. Cavarzere and cols. found a correlation between TSH and birth weight only in babies weighing < 1,000 g (28). In a meta-analysis, Hashemipour and cols. found birth weight to be strongly associated with thyroid function tests (34).

The clinical relevance of THOP, its long-term impact on the neurodevelopmental outcome, and need for replacement of thyroxine require more research, as there is a lack of clarity, despite many studies being conducted in the past. It has still not been possible to implement universal newborn screening for CH in India. Therefore, no universal recommendation can be made regarding rescreening of all preterm/LBW babies. However, high-risk preterm babies, such as those who are extremely LBW and sick preterm babies with prolonged hospital stay, can be subjected to a first screening at 48-72 hours of life, and rescreening can be done at 2 weeks of life.

Further studies from India and other lower- and middle-income countries will help in understanding the problem of THOP and CH with delayed TSH elevation in a better way, as the characteristics of the newborn population in such countries differ from those in developed countries.
